# My home—my castle? Self-reported anxiety varies in relation to the subjective evaluation of home environment

**DOI:** 10.3389/fpsyg.2023.1267900

**Published:** 2024-01-10

**Authors:** Sandra Weber, Anna Mascherek, Jobst Augustin, Bastian Cheng, Götz Thomalla, Hanno Hoven, Volker Harth, Matthias Augustin, Jürgen Gallinat, Simone Kühn

**Affiliations:** ^1^Department of Psychiatry and Psychotherapy, University Medical Center Hamburg-Eppendorf, Hamburg, Germany; ^2^Institute of Health Care Research in Dermatology and Nursing, University Medical Center Hamburg-Eppendorf, Hamburg, Germany; ^3^Klinik und Poliklinik für Neurologie, University Medical Center Hamburg-Eppendorf, Hamburg, Germany; ^4^Institute for Occupational and Maritime Medicine (ZfAM), University Medical Center Hamburg-Eppendorf, Hamburg, Germany; ^5^Lise Meitner Group for Environmental Neuroscience, Max Planck Institute for Human Development, Berlin, Germany

**Keywords:** anxiety, subjective evaluation of home environment, housing, indoor lifestyle, Hamburg City Health Study, mental well-being

## Abstract

**Introduction:**

Although people spend most of the day in their home environment, the focus of research in environmental psychology to date has been on factors outside the home. However, it stands to reason that indoor quality likewise has an impact on psychological well-being. Therefore, the present study addresses the question of whether the subjective evaluation of home environmental parameters are related to self-reported anxiety and whether they can additionally explain variance beyond the usual sociodemographic and general lifestyle variables.

**Methods:**

Data from the Hamburg City Health Study (first 10,000 participants) was analyzed. A subsample of *N* = 8,886 with available GAD-7 anxiety data was selected, and hierarchical regression models were computed, with demographic data entered first, followed by variables concerning lifestyle/habits and finally variables of the subjective evaluation of home environment.

**Results:**

Using the integrated model, we were able to explain about 13% of the variance in self-reported anxiety scores. This included both the demographic, lifestyle, and subjective evaluation of home environment variables. Protection from disturbing night lights, a greater sense of security, less disturbing noises, brighter accommodations, and a satisfactory window view explained almost 6% of the variance and was significantly associated with lower anxiety scores.

**Conclusion:**

The home as a place of refuge plays an increasingly important role as home office hours rise. It is therefore crucial to identify domestic factors contributing to people's mental well-being. The subjective evaluation of one's home environment has proven influential over and above modifiable lifestyle variables.

## 1 Introduction

Environmental psychology is concerned with human-environment interactions and thus also explores the association between environmental factors and human mental well-being. In environmental psychology, environmental spaces can be divided into different levels that range from being rather close or rather far from the individual. Brown (1987) distinguishes between primary, secondary, and tertiary territories. Tertiary territories are accessible to the public without access restrictions (e.g., city squares, sidewalks, etc.), in contrast to this, only certain groups of people have access to secondary territories (e.g., employees who have a key for certain areas) (Flade, 2018). The primary territory (e.g., apartment, house), on the other hand, can be shaped and changed only by the individual in a self-determined way (Brown, 1987). Often, there is a strong emotional attachment, the so-called place attachment, to these personal, highly significant territories (Hidalgo and Hernandez, 2001; Evans et al., 2003).

Generally, one's own home as a primary territory does not only represent a physical space but also a place of psychological significance (Graham et al., 2015). It helps to satisfy basic human needs for control (Smith, 1994) and attachment (Lohmann et al., 2003), as well as being an object of self-expression (Gosling et al., 2002) that contributes to self-esteem enhancement. In this sense, Lewicka refers to one's home as a place with prototypical characteristics (Lewicka, 2011), characterized by many individual qualities, including warmth and privacy (Smith, 1994).

An interesting aspect here is the Personal Boundaries Theory. An explanatory approach, originally to describe the difference of intensity of openness between nurses and their patients (Scott, 1986). The author later expanded her theory to refer more generally to the metaphorical separation between people and their environment (Stiles et al., 2009). In between are the personal boundaries, a kind of filtering device to protect the individual from environmental overload (Scott and Dumas, 1995). This boundary varies from individual to individual and therefore varies in permeability and flexibility (Stiles et al., 2009). For the investigation of the housing variables, this could mean that the participants are influenced to varying degrees by environmental factors, depending on the severity of their filter.

In particular, the fulfillment of the need for security makes the home environment a tremendously important place of refuge (Douglas, 1991). Moreover, it has been shown that the same factors responsible for associating one's home with a recovery process (e.g., calmness, privacy) are also the qualities that lead to evaluating one's home as a secure base, for example, in response to stress (Meagher and Cheadle, 2020). It could be inferred that people feel more connected to their home if it has emotion-regulating capabilities.

During periods of increased worry, such as the COVID-19 pandemic, and limited resources, the home can provide a source of refuge, safety, and stability, even preventing some people from depressive and anxiety-related symptoms (Meagher and Cheadle, 2020). Especially since most important social contacts take place in the home environment (Bronfenbrenner and Evans, 2000) and the living experience is primarily geared toward providing a counterbalance to tension, work, and the fulfillment of duties (Harth and Scheller, 2012). People in Germany spend an average of 15.7 h a day in their home environment (Brasche and Bischof, 2005), and it can be assumed that this has increased significantly during the COVID-19 pandemic. A study in Tokyo, for example, evaluated data from more than 200,000 cell phones and results suggest that 1 week after the lockdown (in 2020), residents' mobility had decreased by 50% and their social contacts by 70% (Yabe et al., 2020), making one's own home all the more important. The complex set of conditions describing housing and immediate living conditions has special significance as people can partly shape it themselves. This is a decisive advantage compared to environmental factors which can only be influenced by individuals to a limited extent, such as industrial and agricultural production sites, which often enough have negative side effects (Hellbrück and Kals, 2012; Hernández et al., 2016; Foster et al., 2022). This means that in times of global challenges with economic consequences and social changes, the home is most likely becoming increasingly important as a safe retreat. Overall, studies show that social contacts decrease in such phases and at the same time the importance of one's own living space increases, especially as it is perceived as controllable.

Studies have specified the general relationship between living conditions and specific clinical pictures. In the context of depression, the amount of daylight at home, air quality, or pest infestation have been examined as objectifiable variables. Other variables are more concerned with subjective factors such as perceived housing safety or noise pollution. Brown and Jacobs (2011) found a relationship between insufficient daylight at home and self-reported as well as physician-diagnosed depression. A study by the World Health Organization identified subjective home satisfaction as a predictor of well-being (World Health Organisation, 2007). These studies show that the immediate home environment and, in particular, its evaluation has measurable effects on residents' well-being. A recent study by Ascone et al. (submitted) explained 16% of the variance in depression scores among a subclinical group (PHQ-9 score < 10) using an integrated model (demographic, lifestyle, subjective evaluation of home environment, and home environment covariates), with more than one-third of the explained variance (6%) accounted for by variables of the subjective evaluation of home environment. However, not only the association between self-reported levels of depression and home environment but also between anxiety and home environment poses an interesting question.

Anxiety disorders are among the most common mental health disorders, with a lifetime prevalence of 33.7% (among adults aged 18–64 years) (Kessler et al., 2005; Bandelow and Michaelis, 2022). Reported anxiety levels increased dramatically during the COVID-19 pandemic. Flanagan et al. (2021) report an increase of nearly 100% with respondents indicating that symptoms affected their daily routines. This makes it all the more important to learn more about aspects of the home environment that are potentially associated with variables describing mental well-being concerning levels of anxiety.

Several studies have suggested links between anxiety disorders and general environmental factors that are beyond the influence of the individual. For example, one study found a significant association between environmental greening and benzodiazepine use in patients diagnosed with anxiety and depression disorders. Interestingly, this association was mediated by air pollution (0.8–29.6%) and noise (2.2–5.3%), whereas physical activity and social support only played a minor role (Gascon et al., 2018).

In a meta-analysis on protective and risk factors for anxiety disorders, the authors point to a notable gap in research identifying factors that might be modified by at-risk individuals and specifically relate to the home environment (Zimmermann et al., 2020). Aspects of the home environment and its subjective evaluation seem to be of particular interest as the identification of these risk factors is considered an essential prerequisite to developing effective prevention strategies (Munoz et al., 2010; Jacka et al., 2013).

The studies cited above are characterized by a substantial amount of heterogeneity, however, they all point to the importance of the association between home environment and health or well-being. With this in mind, the present study aimed to investigate the association between the subjective evaluation of home environment and self-reported levels of anxiety using population data from the Hamburg City Health Study (HCHS).

## 2 Materials and methods

### 2.1 Sample

The data originated from the ongoing Hamburg City Health Study (HCHS), conducted at the University Medical Center Hamburg-Eppendorf. This unique study aims at better understanding the complexity of the interplay between factors, such as the environment, biology, genetics, and lifestyle and health [for details, see Jagodzinski et al. (2020)]. Ethical approval for HCHS was obtained by the Local Ethics Committee of the Landesärztekammer Hamburg.

The data for the present study was collected between 2016 and 2018 and consisted of the first *N* = 10,000 participants of the HCHS. A total of 1114 participants had to be excluded: 978 participants did not provide data on the outcome variable [GAD-7, Spitzer et al. (2006)] and 136 participants scored <24 on the Mini-Mental State Examination [MMSE; Folstein et al. (1975)], indicating potential cognitive impairment. Thus, the sample consisted of *N* = 8886 participants. On average, participants were 62 years old (SD = 8.4; age range between 46 and 78 years), with 51% being female.

### 2.2 Instruments

Anxiety was assessed with the Generalized Anxiety Disorder Scale [GAD-7, Spitzer et al. (2006)]. The seven items were answered on a 4-point Likert scale, ranging from 0 = never to 3 = almost every day. The total score was calculated by using the sum score while using a clinically validated cut-off score of ≥ 10 to describe self-reported levels of anxiety on a level of clinical relevance.

### 2.3 Demographic variables

Age was assessed in years and biological sex as binary variable (0 = male, 1 = female). Income was recorded in 17 ordinal categories. The question “What is your net income per month in euros?” was answered with 1 = <500€/month up to 17 = more than 8,000€/month.

### 2.4 Lifestyle variables

The number of household members including the participant was assessed (min. = 1) as well as health variables (smoking: 0 = non-smoker, 1 = current smoker; alcohol consumption during the last 12 months: 0 = rarely; describing consumption up to 4 times/month, 1 = regularly; describing consumption several times/week) and leisure time activities (TV and computer time: 0 = never, 2 = <1 h/day, 2 = 1–2 h/day, 3 = 2–3 h/day, 4 = 3–4 h/day, 5 = >4 h/day; physical activity: 0 = no regular exercise and 1 = regular exercise).

### 2.5 Home environment covariates and subjective evaluation of home environment

The size of the current home in square meter was assessed as a home environment covariate. Variables of the subjective evaluation of home environment were assessed on a 5-point Likert scale. Variables were: Protection from disturbing nightlight (1 = very poor to 5 = very good; higher score = better protection); brightness (scored 1 = very poor to 5 = very good; higher score = more brightness in the apartment from natural light); perceived safety of apartment (scored 1 = very poor to 5 = very good; higher score mea*n* = higher perceived safety); overall quality of the window view (scored 1 = very poor to 5 = very good; higher score = better quality of window view). Noise disturbance was rated as the mean value of noise during the week, on weekends and at night on a 4-point Likert scale, not taking into account the source of noise (from 0 = not at all to 3 = very much).

### 2.6 Analyses

#### 2.6.1 Preliminary analyses

Missing data. We had complete data for the dependent variable as only cases from the original dataset which had GAD-7 data were included. A differential pattern of missing data emerged for the independent variables. Our sample consisted of 4,316 complete cases, with a total fraction of 7.6% missing data. Missingness per variable varied considerably and ranged from complete data for age and gender to a total of 26.1% for household income, 19.7% for alcohol consumption, and 18.8% for smoking status. The fraction of missing data for all other variables varied between 2 and 6% (see Table 1 for exact numbers of missing data).

**Table 1:** Means and standard deviations for variables under study.

**Variable name**			
	***M***	***SD***	**(*****N*** = **missings)**
Anxiety (GAD-7)	2.8	3.03	
Age in years	62.14	8.42	
Sex^*^	49.3% male	50.7% female	
Income per household category^a^	11.02	3.8	(2,322)
Smoking^*^	82.4 % non-smoking	17.6 % smoking	(1,667)
Alcohol consumption^*^, ^b^	52.7% rarely	47.3% regularly	(1,753)
Use of TV^c^	1.9	0.92	(584)
Use of PC^c^	1.25	0.79	(606)
Physical activity on a regular basis^*^	8.6% no	91.4% yes	(231)
Household size	2.08	0.93	(538)
Size of home (m^2^)	104.38	55.07	(478)
Nightlight	4.32	0.78	(395)
Brightness	4.34	0.76	(371)
Safety	4.23	0.69	(393)
Window view	3.31	0.75	(439)
Noise	0.41	0.53	(500)

Missings per variable are shown in brackets, whole sample N = 8,886.

* Percentage in binary variable.

a Income is subdivided into 17 categories, ranging from below 500€ in category 1 to more than 8,000€/month in category 17. Categories span 250€ each, 11 corresponds to 3,000–3,500€ per month.

^b^ Alcohol consumption during the last 12 months: rarely: describing consumption up to 4 times/month, Regularly: describing consumption several times/week.

c TV and computer time are subdivided into the following categories: never, < 1 h/day, 1–2 h/day, 2–3 h/day, 3–4 h/day, >4 h/day.

In order to account for the potential bias in the data due to missing items, we conducted all of our analyses under a full information maximum likelihood approach (FIIML) in Mplus, where all available data points are included into the analyses, preventing listwise deletion (Muthén et al., 2010). We used MLR estimator for robust standard errors throughout the analyses. We also tested the unadjusted association between our key variables of interest and anxiety in a preliminary step. Because sometimes, the inclusion of variables bears the risk of inducing bias to the analyses instead of controlling it. The analyses of the unadjusted set may be seen as a sensitivity analysis to test this. All variables were significantly associated with anxiety in the unadjusted model, adding to the plausibility of our analyses. Parameter are described in the Supplementary material (see Elwert and Winship, 2014 and Rohrer, 2018, for a comprehensive discussion).

#### 2.6.2 Main analyses

Multiple linear regression analyses were used to run different models to predict self-described anxiety. Variables were entered stepwise so that the potential incremental value could be determined, indicating the potential influence of the home environment over and above the standard demographic variables. The first step included sex, age, and income as predictor variables. The second step included the variables on lifestyle factors and habits: Smoking status, alcohol consumption, number of household members, time spent in front of the TV/computer, and regular physical activity. In the third and final step, the primary variables of interest were added: the subjective evaluation of the home environment (protection from disturbing nightlight, brightness, perceived safety, quality of window view, noise disturbance) and home environment covariates (size of apartment, duration of living in the current dwelling).

In addition, several sub-analyses were performed. First, to test whether the variables were of predictive value for individuals reporting higher levels of anxiety, we estimated a logistic regression with the GAD scores categorized as a binary variable above or below the clinical threshold of GAD sum score >10. Second, interactions with sex, age, and income were tested, since these variables are known influential factors on self-reported anxiety. We proceeded by first testing separate regression models with interaction terms for sex, age, and income. Then, in the next step, variables that showed an effect of *p* < 0.10 were included in a model with all significant interactions (Drewelies et al., 2021). Analyses were performed using R (4.1.1) and Mplus, version 8 (Muthén and Muthén, 2017).

## 3 Results

### 3.1 Regression analyses

Descriptive statistics can be found in Table 1. In our first model, we included the variables age, sex, and income. Table 2 shows the predictors with their standardized coefficients.

**Table 2:** Standardized regression coefficients for anxiety with corresponding *p*-values.

	**Model 1**		**Model 2**		**Model 3**	
**Variable**	***p*** **-value**	**Coef**.	***p*** **-value**	**Coef**.	***p*** **-value**	**Coef**.
Age	**< 0.001**	−0.17	**< 0.001**	−0.16	**< 0.001**	−0.13
Sex: female	**< 0.001**	0.12	**< 0.001**	0.15	**< 0.001**	0.16
Income (Household)	**< 0.001**	−0.13	**< 0.001**	−0.12	**< 0.001**	−0.07
Smoking: yes			0.369	0.01	0.279	0.01
Alcohol: regularly			0.825	−0.01	0.710	−0.002
Household size			**0.006**	0.04	**0.008**	0.04
PC			**< 0.001**	0.05	**0.001**	0.04
Physical activity			**< 0.001**	−0.05	**0.001**	−0.04
TV			**< 0.001**	0.06	**< 0.001**	0.05
Sqm^*^					**0.007**	0.03
Nightlight					**< 0.001**	−0.06
Brightness					**< 0.001**	−0.04
Safety					**< 0.001**	−0.11
Window view					**0.016**	−0.03
Noise					**< 0.001**	0.14
R^2^adjusted	0.060		0.071		0.131	

* Square meter. Bold values indicate significance below *p* < 0.05.

As can be seen, women exhibited higher levels of self-reported anxiety than men, and older age as well as higher income was associated with lower self-reported levels of anxiety. Model I had an adjusted R^2^ of 0.06, indicating 6% of explained variance by our first model.

In model II, we added common lifestyle variables to the analyses. Smoking status and consumption of alcohol remained unrelated to self-reported levels of anxiety. Number of individuals living together and time spent in front of the TV or computer were significantly positively associated with levels of self-reported anxiety, indicating higher levels of anxiety with more time spent in front of the TV and computer as well as larger households. Physical activity on a regular basis was associated with lower levels of self-reported anxiety. The inclusion of the variables led to 1% of additional explained variance (adjusted *R*^2^ = 0.071). Standardized coefficients for model II are depicted in Table 2.

We added our variables of primary interest in a third step, namely size in square meters, protection from disturbing nightlight, perceived safety of apartment, brightness, quality of window view, and noise disturbance. Size in square meters was associated with higher levels of self-reported anxiety. Worse protection from disturbing nightlight was significantly associated with higher levels of self-reported anxiety. Also, the subjective evaluation of one's home environment as safe as well as low levels of disturbing noise were significantly related to lower levels of self-reported anxiety. Satisfaction with window view and brightness were additionally associated with lower levels of self-reported anxiety. Overall, the inclusion of the variables describing the subjective evaluation of home environment added another 6% of explained variance to the model (adjusted *R*^2^ = 0.131). Standardized coefficients are depicted in Table 3.

**Table 3:** Standardized regression coefficients for model with interaction terms.

	**Model with interaction terms^a^**
**Variable name**	**Modell III**
**Demographic variables**	**Coef**.
Age in years	**−0.11**
Sex^*^	**0.14**
Income	**−0.04**
**Lifestyle choices**	
Smoking ^**^	0.01
Alcohol^***^	−0.002
Household size	**0.04**
PC	**0.04**
TV	**0.05**
Physical activity^****^	**−0.04**
**Home environment covariates**	
Sqm	**0.03**
Nightlight	**−0.06**
Brightness	**−0.05**
Safety	**−0.11**
Window view	**−0.03**
Noise	**0.11**
Sex^*^Noise	0.03
Income^*^Noise	**−0.06**
Age^*^Noise	**−0.04**

* With male as reference.

** With non-smoking as reference.

*** With rarely drinking alcohol as reference.

**** With no sports as reference.

a Interactions terms were selected in individual models for age, sex, and income. Interactions with p < 0.1 were then included into the joint model reported here.

Coefficients in bold are significant on p < 0.05.

We then re-estimated our model as a logistic regression to test whether the subjective evaluation of participants' home environment was of predictive value for clinical levels of self-reported anxiety. We found that for self-reported levels of anxiety, only three out of our five key variables were of predictive value, namely perceived safety, brightness, and noise. Parameters are displayed in Table 4.

**Table 4:** Results of the logistic regression reporting odds ratios for the dependent variables: self-reported anxiety above or below clinical threshold of ≥10.

**Variable name**	**Odds ratio**	**95% CI**
**Demographic variables**		
Age in years	**0.004**	**0.001–0.019**
Sex^*^	**1.82**	**1.41–2.35**
Income	**0.00**	**0.00–0.035**
**Lifestyle choices**		
Smoking^**^	0.87	0.62**–**1.22
Alcohol^***^	0.83	0.62**–**1.12
Household size	**1.2**	**1.05–1.37**
PC	**1.18**	**1.02–1.36**
TV	**1.18**	**1.03–1.35**
Physical activity^****^	**0.61**	**0.43–0.87**
**Home environment covariates**		
Sqm	0.90	0.60**–**1.34
Nightlight	0.94	0.81**–**1.1
Brightness	**0.77**	**0.67–0.92**
Safety	**0.75**	**0.63–0.89**
Window view	0.92	0.77**–**1.10
Noise	**1.74**	**1.43–2.11**

* With male as reference.

** With non-smoking as reference.

*** With rarely drinking alcohol as reference.

**** With no sports as reference.

Odds ratios in bold are significant on p < 0.05, which is indicated by the 95% confidence interval not including 1.

In a final step, we also tested for interaction. First, we tested interactions with age, sex, and income in separated models and extracted all interactions on *p* < 0.10 to be re-analyzed in a joint model (Drewelies et al., 2021). Results for the joint model can be seen in Table 3. As this model was part of a set of sub-analyses, it is displayed in a separate table. Younger individuals were more affected by noise disturbance in terms of self-reported levels of anxiety than older individuals. Also, although significantly associated in all groups, the association between noise and self-reported levels of anxiety was stronger in individuals with lower household income. For a detailed description of the interaction effects, see online Supplementary material.

## 4 Discussion

The aim of the present study was to investigate the association between the subjective evaluation of home environment, home environment covariates, and self-reported levels of anxiety in a large sample of middle-aged and older adults in Hamburg, Germany.

Overall, our final model explained roughly 13% of variance, with 6% attributable to subjective evaluation of the home environment. In our sample, more variance was explained by home environment than by common lifestyle variables such as smoking, physical activity, television time, or computer use. This is interesting because generally, lifestyle variables have been studied far more. In the following, we will turn to an in-depth discussion of our findings.

Looking first at income, demographic data, and lifestyle variables, we found that women reported anxiety more frequently than men. This is consistent with the literature, which has shown women to be twice as likely to suffer from anxiety disorders as men and that this distribution is stable across cultures (Bandelow and Schüller, 2020; Bandelow and Michaelis, 2022). There are biological [e.g., Bandelow and Domschke (2015)], psychosocial [e.g., Bandelow et al. (2002)], and societal [e.g., Hurrelmann and Kolip (2015)] explanations for this. Similarly, our study showed that older age and higher income were associated with lower levels of self-reported anxiety. These results are also consistent with epidemiological studies that show lower levels of anxiety in older age [e.g., Bandelow (2003), Kessler et al. (2005), Rubio and López-Ibor (2007a,b)] and lower levels of anxiety in fully employed people with higher income (Jacobi et al., 2004). The lifestyle factors smoking and consumption of alcohol were not related to reported anxiety. Physical activity was associated with lower levels of self-reported anxiety, whereas a positive association emerged for the number of household members, indicating that living together does not *per se* protect against feelings of anxiety.

Looking at our variables of primary interest, the subjective evaluation of home environment and home covariates, variables explicitly describing disturbances, and lower levels of perceived safety were associated with self-reported anxiety. Our results exhibited a significant positive relation between the level of self-reported anxiety and disturbing nightlight and noises disturbances. A negative relation emerged for levels of perceived safety. Perceived quality of window view and brightness were also negatively associated with self-reported levels of anxiety, indicating that brighter accommodations and greater satisfaction with the window view were associated with lower levels of reported anxiety. Given that safety and control are among basic human needs, it stands to reason that variables representing these aspects of the home environment are associated with well-being and, in our study, explicitly with levels of self-reported anxiety.

Disturbing nightlight was significantly associated with higher self-reported anxiety scores. In industrialized countries, exposure to artificial light at night is a widespread phenomenon and light pollution is considered a stressor. A study conducted in Hong Kong (*N* = 369) found that a quarter of respondents felt severely affected by artificial night-time city light. Among other problems, they reported sleep loss and anxiety (Karol et al., 2010). Generally, the disruption of the circadian rhythm can negatively affect behaviour, performance, as well as physiological functions. Several studies have found associations with mood disorders, weight gain, and social problems (Lunn et al., 2017), as well as increased cardiovascular risk under chronic conditions (Grimaldi et al., 2016). Our study suggests that disturbing nightlight is also relevant in a non-clinical sample with respect to reported feelings of anxiety. While our study described nightlight as a relevant source of disturbance for levels of anxiety, future studies should take a closer look at specific aspects of nightlight, e.g., type and duration of light disturbance to further understand the underlying mechanisms, especially in healthy individuals.

Higher levels of perceived safety were associated with lower levels of self-reported anxiety. This is in line with the literature, where a recent British study (*N* = 9,205) showed that safety was associated with happiness and lower levels of anxiety (Huebner et al., 2022). The need for security represents an important aspect, only exceeded by basic physiological needs (Maslow, 1954). It has been shown that satisfied needs for security and protection in one's own living space are associated with higher quality of life (Grütter, 2021). Hence, safe living environments might contribute to locus of control which is positively associated with health. However, the subjective perception of safety also depends on the respective emotional, physical, and social resources of a person (Bals, 2004), resulting in interindividual differences (Klimke, 2008). The results of our study underline that subjectively perceived safety represents an important aspect of well-being and mental health.

Noise disturbance emerged as another aspect of the home environment significantly related to self-reported levels of anxiety. We used an aggregated measure of noise exposure across time of day and source of noise. In our study, lower overall self-reported noise disturbance was associated with lower levels of self-reported anxiety. Our results are in line with recent literature and underline the problematic association between noise pollution and well-being. In an Australian study, frequency of noise exposure significantly predicted levels of anxiety in 2065 participants, even when the effects of both neighborhood affiliation and demographic covariates were taken into account. Participants who suffered from frequent noise exposure were more anxious (Bower et al., 2023). Another study from the UK examined changes in how homes were inhabited and how the intended purpose of a home changed, housing quality, and associated well-being among London residents (*n* = 1,250) during the COVID-19 pandemic. 37.9% of respondents felt adversely affected by housing conditions, with noise being the most common problem and the most influential factor (Jacoby and Alonso, 2022). A meta-analysis including five studies with a total of *n* = 372,079 participants found a 12% (95% CI: −4%, 30%) higher likelihood of anxiety, which was proportionally associated with a 10 dB(A) increase in night-time noise levels during the day and evening (Dzhambov and Lercher, 2019). Hence, our study, confirms findings from previous studies that acknowledge the harmful effect of noise on well-being.

Brightness and quality of window views were associated with lower levels of self-reported anxiety, for which we present the following explanation. It has been suggested that light conditions play a crucial role for humans, with brightness linked to an unrestricted possibility to see (prospect) and darkness linked to the possibility to hide (refuge) (Appleton, 1996). Living in brighter apartments might therefore also be associated with a greater feeling of safety. This remains speculative as participants did not describe their actual window view but only their satisfaction with it, it seems plausible that window views leading to more satisfaction might have been unobstructed. This would fit into Appleton's notion of brightness being associated with the possibility to see and to evaluate the surroundings. We ask the reader to be cautious when interpreting the results, but consider this an interesting route to follow for future studies.

The results of our study generally suggest that it is important to take the home environment and its subjective evaluation into account, as it is significantly related to levels of self-reported anxiety in adults of middle age and older. Interestingly, our results suggest this rather neglected aspect might prove equally important as the more frequently researched lifestyle variables. Thus, we conclude that future studies should take home environmental factors into account to further understand underlying mechanisms and the contribution of aspects of the home environment to individual well-being.

Our study has some limitations which we describe below. First, our results cannot be interpreted causally as this is typical for cross-sectional studies. Longitudinal studies are needed to shed light on our results from a causal perspective. Second, self-selection of participants might well have taken place, because our sample was healthier than average in terms of self-reported anxiety, and the cut-off value in the GAD-7 was exceeded less often than the 12-month prevalence would suggest. This fits to the results of the sub-analyses on whether or not the key variables were of predictive value for self-reported levels above the clinical threshold. In this analysis, only three out of the five variables proved significant, however, this might as well represent an aspect of either power or might be directly linked to the aspect of self-selection mentioned above. Having an overall healthier sample than the average in terms of self-reported anxiety, leaves room for speculations concerning potential idiosyncrasies of the rather small subgroup reporting anxiety levels above the clinical threshold. However, this question cannot be answered from the results of the present study, it should, however, caution the reader against jumping to a general conclusion. Third, it remains an open question of how the association between levels of self-reported anxiety and subjective evaluation of home environment was built. From our data, two lines of interpretation are equally conceivable: A less favorable home environment might be associated with higher levels of self-reported anxiety as discussed above. Anxiety levels might also influence the perception of the environment and thus also the evaluation of the home environment variables assessed in the present study. The general question of causation remains unanswered form the present study. Individuals living under poorer conditions might show lower levels of wellbeing and might even develop mental illnesses out of deprivation. However, higher levels of anxiety and tension might hinder individuals to take jobs or other opportunities that would enable better housing and living conditions. The results of our study are not suitable to interpret the findings in either of the three directions. However, the results point to the importance of the evaluation of the subjective home environment as one aspect of assessing levels of anxiety.

Although we have no data on this, it is also conceivable that self-reported anxiety is dependent on compliance with individual, personal boundaries. The more a resident feels that their boundaries are respected in their living environment, the better they can identify with their surroundings and the safer they feel.

Generally, perception is characterized by a necessary selection of information to prevent overstimulation of the brain. Thus, self-reported information always represents a subset of objectively available information, biased by personal experiences, needs, or one's own self-concept. This attentional bias results in a very individual, selective representation of objective realities. This could imply that the more anxious individuals in our study reported aspects of the home environment differently resulting in an inextricable link between anxiety levels and perceived environment. To shed light on this potential bias, future studies could benefit from also including objective measures of the home environment.

To conclude, the results of the present study suggest a relationship between the subjective evaluation of home environment and levels of self-reported anxiety. Perceived safety, noise disturbance, brightness, quality of window view, and disturbing nightlight explained 6% of variance, indicating that the home environment represents an important aspect of subjective well-being. Future studies are needed to further investigate the underlying mechanisms and to shed light on causal mechanisms.

## Data availability statement

The datasets for this study belong to the ongoing HCHS study and therefore cannot be provided by the authors. Requests to access the datasets should be directed to Epidemiologisches Studienzentrum: hchs@uke.de.

## Ethics statement

The studies involving humans were approved by the Local Ethics Committee of the Landesärztekammer Hamburg. The studies were conducted in accordance with the local legislation and institutional requirements. The participants provided their written informed consent to participate in this study.

## Author contributions

SW: Conceptualization, Writing—original draft. AM: Formal analysis, Writing—review & editing. JA: Writing—review & editing. BC: Writing—review & editing. GT: Writing—review & editing. HH: Writing—review & editing. VH: Writing—review & editing. MA: Writing—review & editing. JG: Writing—review & editing. SK: Conceptualization, Supervision, Writing—review & editing.
