# Supplementary material for: My home—my castle? Self-reported anxiety varies in relation to the subjective evaluation of home environment
Source: Front Psychol. 2024 Jan 10;14:1267900. doi: 10.3389/fpsyg.2023.1267900 (PMC10806144; doi:10.3389/fpsyg.2023.1267900)
Supplement: Supplementary file 1 [file Data_Sheet_1.PDF]

Supplementary Material

We show interaction plots and results of the simple slope analyses for variables that resulted in a significant interaction effect in our full interaction model (see manuscript).

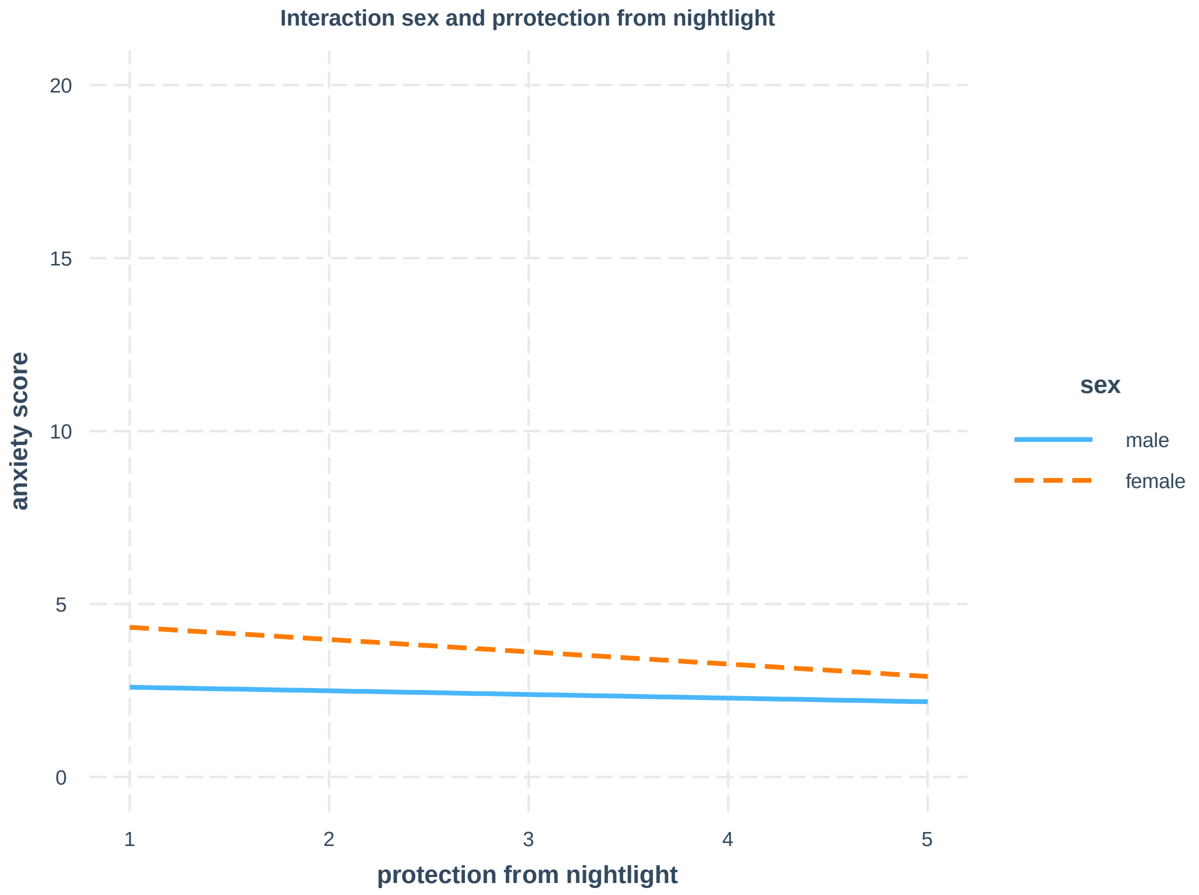

Supplementary Figure 1. Interaction between sex and protection from nightlight.

Supplementary Table 1. Simple slope analysis for interaction between protection from disturbing nightlight and sex.

	Estimate (s.e.)	95% CI
female	**-0.36** (0.08)	-0.51 – -0.20
male	-0.11 (0.08)	-0.26 – 0.05

s.e. = standard error of the estimate; 95% CI = upper and lower bound of the 95% confidence interval. Estimates in bold indicate significance on $p < .05$.

Figure 1 and table 1 further illustrate the interaction effect. Men and women differ significantly in their evaluation of the effect of protection from disturbing nightlight and levels of self-reported anxiety. The effect of reported protection from disturbing nightlight is significantly related to self-reported levels of anxiety only in women.

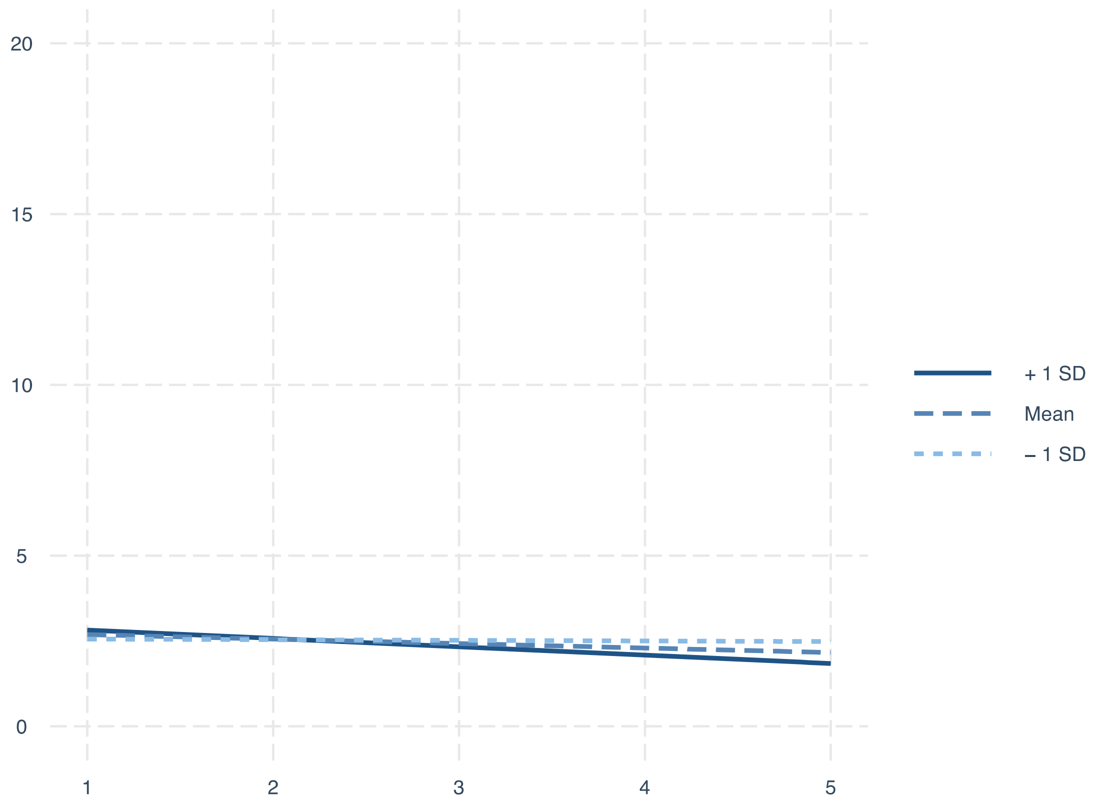

Supplementary Figure 2. Interaction between brightness and household income.

Supplementary Table 2. Simple slope analysis for interaction between brightness and household income.

	Estimate (s.e.)	95% CI
1 SD below the mean	-0.02 (0.08)	-0.17 – 0.13
mean	**-0.13** (0.06)	-0.25 – -0.01
1 SD above the mean	**-0.24** (0.08)	-0.41 – -0.08

SD = standard deviation; s.e. = standard error of the estimate; 95% CI = upper and lower bound of the 95% confidence interval. Estimates in bold indicate significance on $p < .05$.

Figure 2 and Table 2 further illustrate the interaction effect. We now report the interaction effect for the manifestation of household income for 1 standard deviation below the mean, the mean itself, and 1 standard deviation above the mean household income. We can see that the association between brightness and self-reported levels of anxiety varies significantly between different household income categories. However, the effect on anxiety levels is only significantly different from zero in mean level income groups and groups with income above the mean. Brightness is not significantly related to self-reported levels of anxiety in groups well below the mean.

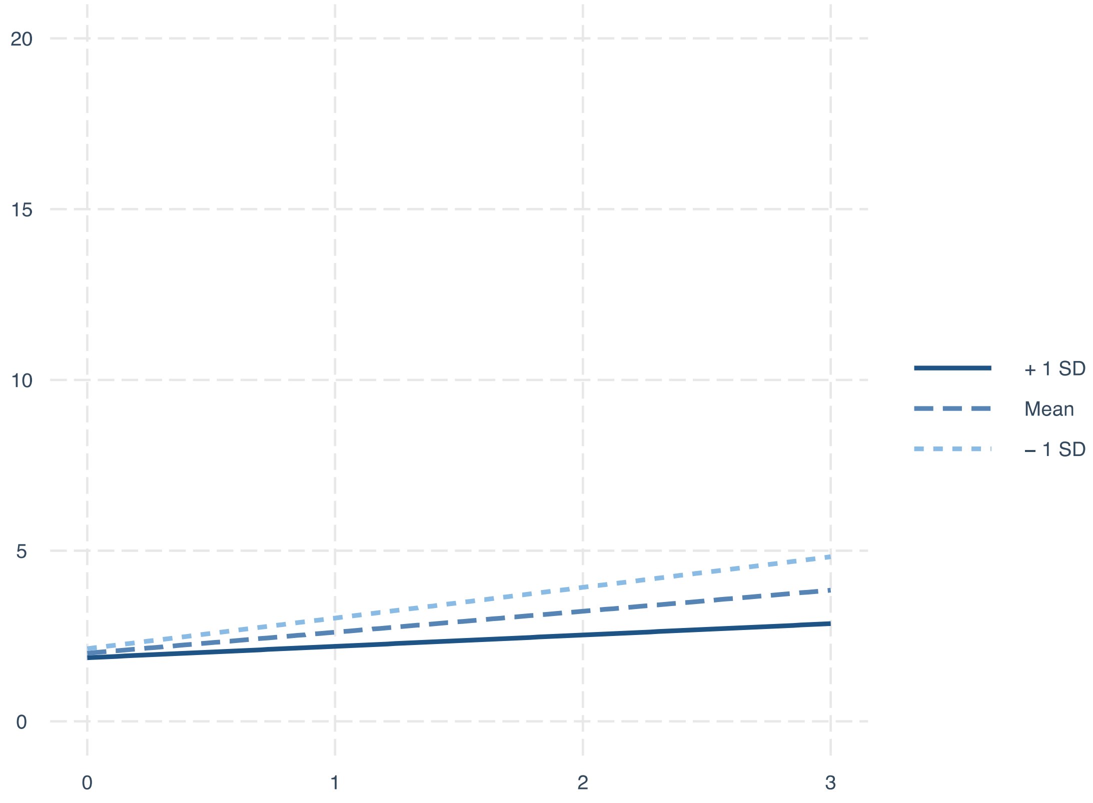

Supplementary Figure 3. Interaction between noise and household income.

Supplementary Table 3. Simple slope analysis for interaction between noise and household income.

	Estimate (s.e.)	95% CI
1 SD below the mean	**0.90** (0.10)	0.70 – 1.10
mean	**0.61** (0.08)	0.45 – 0.78
1 SD above the mean	**0.33** (0.12)	0.09 – 0.57

SD = standard deviation; s.e. = standard error of the estimate; 95% CI = upper and lower bound of the 95% confidence interval. Estimates in bold indicate significance on $p < .05$.

Figure 3 and Table 3 further illustrate the interaction effect. We now report the interaction effect for the manifestation of household income for 1 standard deviation below the mean, the mean itself, and 1 standard deviation above the mean household income. We can see that the association between disturbance by noise and self-reported levels of anxiety is differently pronounced in different household income categories. The effect on anxiety levels is only strongest income groups below the mean and weakest in groups above the mean. However, it is significantly different from zero in all three groups.

We tested the unadjusted association between our key variables of interest and anxiety in a preliminary step. Because sometimes, the inclusion of variables bears the risk of inducing bias to the analyses instead of controlling it. The analyses of the unadjusted set may be seen as a sensitivity analysis to test this. All variables were significantly associated with anxiety in the unadjusted model, adding to the plausibility of our analyses (see Elwert & Winship, 2014 for a comprehensive discussion).

Supplementary Table 4. Regression coefficients, standard errors and confidence intervals for each variable of the subjective evaluation of the home environment separately (separate analyses) and in a model with only the key variables of interest (model key variables).

		Separate analyses			Model with key variables only	
Variable	Coef.	Std Error	Conf. Int (95%)	Coef.	Std. Error	Conf. Int (95%)
nightlight	**-0.66**	0.05	-0.75 – -0.57	**-0.27**	0.05	-0.36 – -0.17
brightness	**-0.58**	0.05	-0.67 – -0.48	**-0.20**	0.05	-0.34 – -0.1
safety	**-0.81**	0.05	-0.91 – -0.70	**-0.48**	0.06	-0.62 – -0.37
Window view	**-0.64**	0.05	-0.74 – -0.55	**-0.18**	0.06	-0.32 – -0.07
noise	**1.18**	0.07	1.04– 1.33	**0.83**	0.08	0.6 – 0.98

Note: estimates printed in bold indicate $p<0.05$.

Elwert, F., & Winship, C. (2014). Endogenous selection bias: the problem of conditioning on a collider variable. Annual Review of Sociology, 40(1), 31-53. https://doi.org/10.1146/annurev-soc-071913-043455
